# Increase in PKCα Activity during Heart Failure Despite the Stimulation of PKCα Braking Mechanism

**DOI:** 10.3390/ijms21072561

**Published:** 2020-04-07

**Authors:** Naveed Aslam

**Affiliations:** 1 BioSystOmics 4424 Jim West Street, Bellaire, TX 77401, USA; naveed.aslam@biosystomics.com; 2 Department of Chemistry & Chemical Engineering, Lahore University of Management Sciences (LUMS), Lahore 54792, Pakistan

**Keywords:** myocardial infarction, translocation, lipid, contractile dysfunction, β-adrenergic system desensitization

## Abstract

Rationale: Heart failure (HF) is marked by dampened cardiac contractility. A mild therapeutic target that improves contractile function without desensitizing the β-adrenergic system during HF may improve cardiac contractility and potentially survival. Inhibiting protein kinase C α (PKCα) activity may fit the criteria of a therapeutic target with milder systemic effects that still boosts contractility in HF patients. PKCα activity has been observed to increase during HF. This increase in PKCα activity is perplexing because it is also accompanied by up-regulation of a molecular braking mechanism. Objective: I aim to explore how PKCα activity can be increased and maintained during HF despite the presence of a molecular braking mechanism. Methods and Results: Using a computational approach, I show that the local diacylglycerol (DAG) signaling is regulated through a two-compartment signaling system in cardiomyocytes. These results imply that after massive myocardial infarction (MI), local homeostasis of DAG signaling is disrupted. The loss of this balance leads to prolonged activation of PKCα, a key molecular target linked to LV remodeling and dysfunctional filling and ejection in the mammalian heart. This study also proposes an explanation for how DAG homeostasis is regulated during normal systolic and diastolic cardiac function. Conclusions: I developed a novel two-compartment computational model for regulating DAG homeostasis during Ang II-induced heart failure. This model provides a promising tool with which to study mechanisms of DAG signaling regulation during heart failure. The model can also aid in identification of novel therapeutic targets with the aim of improving the quality of life for heart failure patients.

## 1. Introduction

Shortness of breath and angina are may be the first significant clinical symptoms of coronary artery disease (CAD) [1]. CAD may lead to myocardial infarction (MI). MI can induce structural remodeling [2] in both the left and right ventricles, though it affects the left ventricle more frequently. Clinically, cardiac remodeling manifests through changes in the size, shape, and function of the heart [2,3]. Remodeling events are characterized by complex architectural alterations that result in rearrangement of the existing myocardial structure. These changes take place at both infarcted and non-infarcted areas of the myocardium [2,3]. These alterations include dilation of the ventricular cavity, myocyte necrosis, thinning of the infarcted myocardium, and interstitial fibrosis [1,2,3,4,5]. After MI, early ventricular remodeling is a compensatory adaptive response to loss of function in the myocardium. The architectural alterations following MI can, in turn, preserve cardiac performance, at least in the early phase immediately following infarction. However, these initial compensatory changes can transition into permanent modifications of the cardiac structure leading to heart failure [1,2,3,4,5]. Despite significant progress in our understanding of the cellular and molecular basis of cardiac remodeling, the molecular mechanisms responsible for the transformation of initial compensatory responses into responses that impair cardiac function remain elusive [5].

The transition from compensatory response to loss of function can partially be linked to impaired calcium (Ca^+2^) homeostasis [5,6]. Clinically, dysregulation of calcium signaling manifests as contractile dysfunction and the development of malignant ventricular arrhythmia [5,6]. Experimental observations in angiotensin II (Ang II)-stimulated cardiomyocytes show that, in part, calcium homeostasis is regulated through the Gαq–DAG–PKCα–DGKζ signaling cascade [5,6,7]. PKCα is a key molecule linked to Gαq-induced heart failure [8]. PKCα belongs to the conventional protein kinase C (cPKC) family of serine/threonine protein kinases. These kinases are canonically activated by Ca^+2^ and lipid signaling [9]. PKCα is the most prominent member of the PKC family and is expressed in mouse, human and rabbit heart tissue [8,9]. Previous observations have linked PKCα to impaired left ventricular filling and ejection during heart failure. PKCα is necessary and sufficient to induce ventricular systolic and diastolic dysfunction [8,9]. Pharmacological and genetic inhibition of PKCα clearly improve contractility during heart failure, attenuating the extent of damage and disease [8,9]. Prolonged activation of PKCα may lead to serious malignant outcomes by inducing systolic and diastolic dysfunction [8,9,10,11,12].

DGKζ is another key functional effector that signals during Gαq-induced heart failure [13]. Previous research has shown that cardiac-specific overexpression of DGKζ suppresses remodeling and fibrosis in the left ventricle (LV) independent of hemodynamic regulation. Thus, overexpression of DGKζ under these conditions rescues angiotensin-induced congestive heart failure [13]. Previous data indicates that overexpression of DGKζ also improves survival after MI [14]. Both DGKζ and PKCα have been individually linked to angiotensin II/Gαq-induced cardiac dysfunction, though expression of the two molecules results in opposite outcomes [13,14,15,16]. PKCα appears to be a negative regulator, whereas DGKζ appears to positively regulate the preservation of left ventricular function after MI [9,10,11,12,13,14,15,16]. Some observations indicate that DGKζ can prevent angiotensin II-induced activation of PKCα and subsequent contractile dysfunction [13,14,15,16,17,18]. The precise molecular details of the cardiac-specific protective function of DGKζ, however, remains elusive. Recent experimental evidence from multiple cell types indicates that both PKCα and DGKζ can organize themselves into a local DAG-modulated signaling complex [19,20]. This DAG-based signalosome is instrumental in regulating the duration and amplitude of DAG in response to a variety of stimuli. Formation of this DAG signaling complex is the key to efficient DAG signaling transduction and localization to restricted subcellular sites. PKCα and DGKζ are thought to have opposing functions in this signaling cascade. PKCα acts as a positive regulator whereas DGKζ act as a negative regulator of DAG signaling [19,20].

Human heart failure is linked to increased PKCα activity [12]. This linkage is surprising, as PKCα activity in most cell types is exquisitely regulated [19,20]. PKCα activity in cardiomyocytes is also tightly regulated through a molecular braking mechanism. As soon as PKCα is activated, the molecular brakes in place to check this increase in PKCα concentration should also kick in, acting on the common activator. This molecular braking mechanism begs the question: why is PKCα activity increased and maintained during heart failure despite the concurrent activation of a braking mechanism? Here, I propose an explanation based on data analyzing a local DAG signaling cascade that may be responsible for the increase in PKCα activity during HF.

The purpose of this study is to investigate how positive and negative effector molecules interact with each other during Gαq signaling in the context of angiotensin II-induced heart failure. The rationale for this study is derived from the following experimental observations: (1) Cardiac-specific overexpression of DGKζ improves survival during HF by attenuating LV remodeling [14]. (2) DGKζ prevents translocation of PKCα from the cytosol to the plasma membrane in response to angiotensin-II induced activation [13,14]. (3) DGKζ and PKCα can organize themselves into a local signaling complex [19,20]. (4) PKCα overexpression negatively affects contractility [9,10,11,12]. (5) PKCα down-regulation prevents fibrosis and subsequent alteration of cardiac structure [11,12,13,14]. (6) PKCα is activated in a precise spatiotemporal manner [19,20]. (7) The activation of PKCα depends on the availability of DAG, a tightly-regulated signaling lipid [10,19,20]. (8) A local DGKζ–PKCα signaling complex terminates DAG signaling by converting DAG to phosphatidic acid (PA). PA, in turn, negatively regulates PKCα activity [19,20]. (9) PKCα can induce its own activation by phosphorylating and deactivating DGKζ [21]. These observations suggest that DAG signaling in cardiomyocytes is regulated through a complex regulatory structure.

I propose DAG signaling homeostasis during Ang II-induced heart failure can be regulated through a molecular loop between the positive and negative effector molecules PKCα and DGKζ. This proposed molecular loop may improve understanding of the molecular mechanisms involved in the complex spatiotemporal organization of DAG–PKCα–DGKζ signaling during post-MI cardiac remodeling events. Using a computational model, this study shows the proposed molecular loop has a dual regulatory character. During basal conditions, a net negative feedback loop may prevail and regulate local DAG concentration. Interestingly, upon stimulation conditions, a positive feedback effect on DAG signaling is observed. This positive feedback can possibly explain the link between persistently high DAG levels and malignant outcomes. The transition from negative to positive feedback depends on the local biosynthesis rate of DAG and, in turn, on the mutual interactions between positive and negative DAG effector molecules.

The results of this study imply that a complex sequence of molecular events may regulate DAG homeostasis in a precise, spatiotemporal manner. This regulation includes DAG-induced translocation of PKCα and DGKζ from the cytosol to the plasma membrane, formation of a local signaling complex between PKCα and DGKζ, and conversion of DAG to PA through the action of this signaling complex. Then, the complex is de-stabilized and PKCα and DGKζ return to the cytosol. At the molecular level, these complex events generate a net negative feedback effect on DAG signaling. My computational model indicates that, after MI, DAG homeostasis is likely disrupted due to a positive feedback signal. My model shows the origin of a post-MI positive feedback loop is complex. It is likely modulated through a sequence of molecular events including DAG-induced activation of PKCα, dissociation of the PKCα–DGKζ signaling complex, and PKCα mediated phosphorylation of DGKζ. After these events, it includes translocation of active PKCα from the cell membrane to the cytosol, and deactivation of active PKCα in the cytosol.

## 2. Results

### 2.1. Local DAG Signaling Regulation in Cardiomyocytes

**Figure 1:**
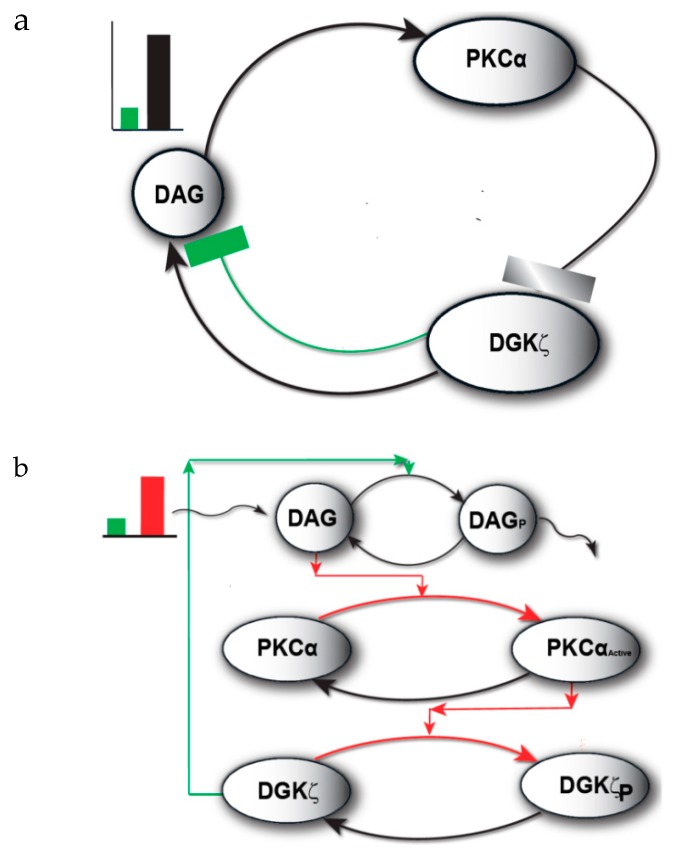
The regulatory molecular loop between the target protein kinase C α (PKCα) and attenuator diacyl glycerol kinase ζ (DGKζ) of local diacylglycerol (DAG) signaling. This regulatory loop explains how the second messenger, DAG, is tightly and dynamically regulated through the formation of a DAG-induced DGKζ–PKCα signaling complex. (**a**) Conceptual diagram of a DAG regulatory loop. This wiring diagram shows that during stimulation levels (green pulse) of low intensity (normal physiological state), DAG homeostasis is regulated through a negative feedback signal. However, at higher levels of stimulation (black pulse; possible pathological state), DAG homeostasis is disrupted due to a positive feedback effect. The positive feedback on DAG signaling is due to DAG-induced activation of PKCα which, in turn, leads to dissociation of signaling complex and subsequent deactivation of DGKζ through phosphorylation. (**b**) Detailed description of the DAG regulatory loop. Here, during normal state (green pulse and green feedback line), the active DGKζ molecule phosphorylates DAG, thereby preventing the PKCα activation. On higher levels of stimulation (red pulse and red feedback line), the local DAG levels increase so sharply that it outpaces the metabolizing ability of the DGKζ–PKCα signaling complex. Thus, net local accumulation of DAG activates PKCα, causing the dissociation of the signaling complex. The active PKCα, in turn, phosphorylates and deactivates DGKζ. This loop also shows that, once active, the PKCα molecule can stimulate its own activation through persistent positive feedback, which can be linked with blunted contractility in heart failure.

The model I propose in Figure 1 describes local regulation of DAG homeostasis in cardiomyocytes. The model is composed of two molecular components: 1. PKCα, the target protein of DAG signaling, can exist in one of four states: cytosolic dormant (PKC_II_α), inactive membrane (PKC_I_α), active membrane (PKC_I_α^Active^) or active cytosolic (PKC_II_α^Active^). 2. DGKζ, the attenuator protein of DAG signaling, can be in one of three states: cytosolic (DGK_II_ζ), active membrane (DGK_I_ζ), or phosphorylated/inactive membrane (DGK_I_ζ_P_). Both these components migrate to the plasma membrane in a DAG-dependent manner. Once in the plasma membrane, DGKζ forms complex C_1_ with PKCα. These two components interact in a closed loop and regulate DAG homeostasis in a negative feedback loop. Here, I assume that, during pathological conditions, complex C_1_ is activated by DAG binding to PKCα. Once active, C_1_ becomes unstable and dissociates into its components DGK_I_ζ and PKC_I_α^Active^. In turn, active PKC_I_α^Active^ phosphorylates and deactivates DGK_I_ζ. My model assumes that, once active, PKCα can stimulate its own activation through a persistent positive feedback loop. This positive feedback may be linked to heart failure [8,9,10,11,12,13,14,15].

**Figure 2:**
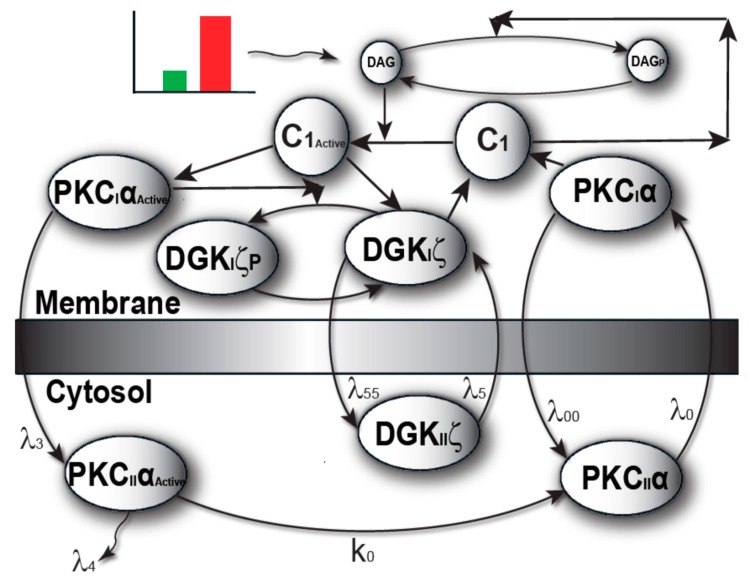
The two-compartment regulatory model of local DAG signaling in cardiomyocytes. Here, one of the compartments is cytosol, whereas the other compartment is plasma membrane. This model explains the spatial and temporal regulation of DAG–PKCα–DGKζ signaling in Gαq-induced heart failure. This model describes a sequence of complex events involving the translocation, activation, association/dissociation, phosphorylation/deactivation and redistribution of target and attenuator molecules of DAG signaling in cardiomyocytes. Here, the PKCα molecule (DAG target) exists in four states: (1) dormant PKCα enzyme residing in cytosol (PKC_II_α); (2) active PKCα enzyme residing in cytosol (PKC_II_α^Active^); (3) inactive molecule residing in the plasma membrane (PKC_I_α); (4) active molecule residing in the plasma membrane (PKC_I_α^Active^). Similarly, the attenuator of DAG signaling, i.e., DGKζ, exists in three forms: (1) enzyme in cytosol compartment (DGK_II_ζ); (2) DGKζ molecule in the plasma membrane (DGK_I_ζ); (3) phosphorylated and inactive molecule residing in the membrane compartment (DGK_I_ζ_P_). During the normal conditions (green pulse), when DAG levels are low, both PKC_II_α and DGK_II_ζ migrate to the plasma membrane in a DAG-dependent manner. Once at the plasma membrane they form a complex C_1_, thus, metabolizing the DAG through phosphorylation. On high intensity stimulation (red pulse), higher levels of DAG are generated, thus stimulating the migration of larger quantities of PKCα and DGKζ from cytosol to membrane. In this case, the local DAG levels increase so sharply that the ability of complex C_1_ to metabolize DAG to PA is outpaced by generation, leading to a transient increase in local concentrations of the second messenger. In turn, this leads to the activation of complex C_1_ through the DAG binding with PKCα. Upon activation, the complex C_1_^A^ becomes unstable and dissociates into PKC_I_α^Active^ and DGK_I_ζ. The active enzyme PKC_I_α^A^ phosphorylates and deactivates the DGK_I_ζ molecule. The active molecule PKC_I_α^Active^ migrates back to cytosol. Once in cytosol, the active form of PKCα enzyme could either degrade or deactivates into its dormant form and is stored for the next translocation–activation–re-translocation cycle. Through a complex process, the inactive DGK_I_ζ_P_ molecule is dephosphorylated, thus, again, forming a complex C_1,_ and eventually reducing the increase in DAG levels and restoring its homeostasis in the plasma membrane.

The model of local DAG signaling (Figure 2) assumes that PKCα can reside in four distinct states. The first is an inactive, dormant state that cannot phosphorylate substrates and resides only in the cytosol. Second, PKCα may reside in an inactive state that cannot phosphorylate substrates and resides only in the plasma membrane. Third, PKCα may exist in an active state obtained when inactive PKCα binds to DAG at the plasma membrane compartment. This active state can phosphorylate other substrates including DGK_I_ζ. Last, PKCα may exist in an active state in the cytosol. This active state is obtained when active PKCα translocates from the plasma membrane back to the cytosol [11,22]. This form of PKCα is prone to degradation. If PKCα does not degrade, it becomes deactivated, and exists as the dormant form of α-enzyme. This active state can also phosphorylate other substrates. The faithful translocation, activation, and re-translocation/de-activation model of PKCα may be more complex than the four-state model assumed here [11]. A more complex model may consider the exact details of the molecular events involved when PKCα translocates from the cytosol to the plasma membrane. The model must also take into account the inhibition of PKCα translocation by DGKζ, as well as enzyme anchoring at the plasma membrane, re-translocation of active PKCα to the cytosol, PKCα degradation, and deactivation. These processes are complex but have been approximated within our model by simpler biochemical kinetic events. These simplified kinetic events preserve key qualitative features of the biochemical interactions within the model. In the computational simulations, PKCα translocation from the cytosol to the plasma membrane is assumed to be regulated through a kinetic event that is directly proportional to the DAG concentration at the plasma membrane. It is assumed the DAG concentration is inversely proportional to the concentration of DGKζ. I also assumed there were non-negligible basal levels of dormant cytosolic PKCα (10 pg/mL) and DGKζ (10 pg/mL). In contrast, the basal concentration of all other forms of PKCα and DGKζ are assumed to be negligibly small. The biochemical reaction rates for the DGKζ–PKCα molecular loop have been published in previous work [19,20,21]. In order to determine the degradation rates of PKCα, constants with experimentally measured rates were used [22].

DGKζ inhibits DAG translocation in my proposed model. Upon stimulation, local generation of DAG induces both PKCα and DGKζ translocation. Once in the plasma membrane, both the DAG signaling target and attenuator proteins form biochemical complex C_1_. This complex, in turn, stimulates the degradation of second messenger DAG, thus restoring homeostasis. In the proposed model, the rate of DAG generation is counterbalanced by a negative feedback effect generated through C_1_-mediated DAG phosphorylation. Under normal physiological conditions, DAG concentration is regulated in this tight spatiotemporal manner. 

The model assumes that during pathological conditions the local rate of DAG generation far exceeds the rate of C_1_-mediated removal. This dynamic leads to a transient increase in local DAG concentration. Local accumulation of DAG, in turn, activates C_1_ through binding PKCα. Once active, C_1_ dissociates into PKC_I_α^Active^ and DGK_I_ζ. Active PKC_I_α^Active^ deactivates DGK_I_ζ through phosphorylation. In this model, PKCα stimulates its own activation through a persistent positive feedback loop. Prolonged PKCα activation may be linked to systolic and diastolic dysfunction. Although the model represents a simplified version of Gαq/DAG signaling, it captures the essential features of local DAG signaling in cardiomyocytes. This local DAG signaling loop is described by a set of biochemical reactions (Equations (1)–(16)).

Local DAG generation in response to angiotensin II-mediated activation of the GPCR pathway is a complex regulatory process [7,9]. GPCR agonist angiotensin II stimulates phospholipase C-mediated hydrolysis of phosphatidylinositol 4,5-biphosphate to produce inositol triphosphate and DAG [7]. Although this study primarily focuses on the downstream signaling of the angiotensin II-stimulated GPCR pathway, Local DAG biosynthesis is modeled using a brief stimulatory pulse that varies in intensity. This pulse mimics the effects of GPCR agonist angiotensin II. This simple approach ignores the finer details of angiotensin II-induced DAG biosynthesis [7]. As the primary focus of this study is how DAG homeostasis is regulated, not DAG biosynthesis, the simple approach was chosen so as not to obscure any overarching results with fine details.

### 2.2. PKCα and DGKζ Translocation in Cardiomyocytes

**Figure 3:**
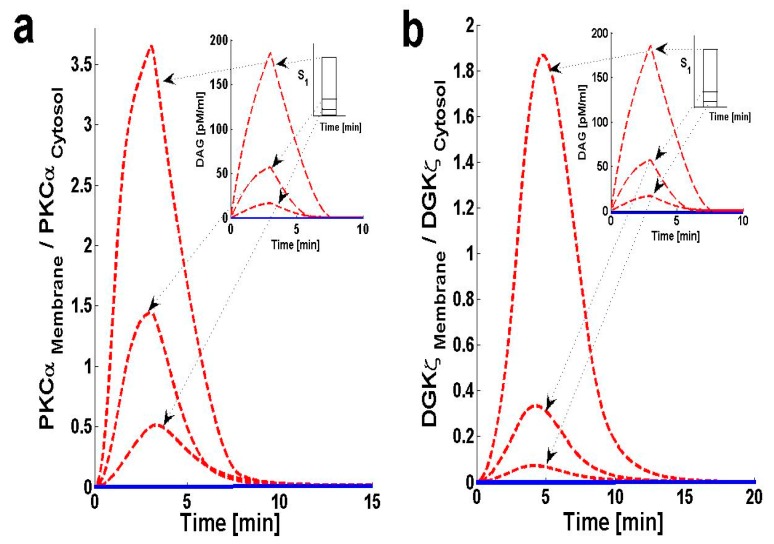
Membrane-to-cytosol (M/C) ratios of target and attenuator molecules of DAG signaling. These results show that pulse-like (duration of 3 min and three different levels of pulse strength are used i.e., “S_1_” = 0.5,2 and 6) stimulation leads to the rapid generation of DAG. The generation of second messenger, in turn, stimulates the translocation of both the target and attenuator molecules of DAG signaling from cytosol to membrane. Here, the solid line represents the non-stimulation condition, whereas the dashed line represents stimulation. (**a**) M/C ratios of PKCα with respect to different levels of pulse-like stimulation mimicking the GPCR agonist angiotensin II (Ang. II). These results show that Ang. II like stimulation leads to rapid de-novo generation of DAG, which, in turn, stimulates the translocation of both PKCα and DGKζ from cytosol to membrane. Here, the translocation rates are set as linear functions of DAG concentration. At low stimulation levels only a small amount of DAG is generated at the plasma membrane thus, inducing the migration of only a small pool of PKCα to the membrane. At the membrane, PKCα forms a biochemical complex C_1_ with DGKζ and stimulates the DAG conversion to PA. Once DAG homeostasis is restored, the complex C_1_ decomposes into DGK_I_ζ and PKC_I_α molecules; these, in turn, quickly re-translocate to the cytosol compartment. At higher stimulation levels, a much larger quantity of DAG is generated, thus stimulating the translocation of a much larger pool of PKCα from cytosol to membrane. High-intensity stimulation leads to a much larger M/C ratio of PKCα and enhanced residence time in membrane compartment. (**b**) M/C ratios of DGKζ with respect to different levels of stimulation. The DGKζ M/C ratio rapidly increases due to a sharp increase in DAG concentration on stimulation. Here, the translocation event of DGKζ is also modeled as a linear proportional relationship to the free concentration of DAG.

Using the computation model, I next determined the translocation characteristics of both the target and attenuator molecules of DAG signaling in cardiomyocytes. In the model, translocation characteristics were determined by measuring the membrane-to-cytosol (M/C) ratio of target and attenuator molecules. The M/C ratio describes the relative distribution of PKCα and DGKζ molecules in the membrane and cytosolic compartments. For PKCα, this ratio is an indirect index of activation. According to the proposed model, the M/C ratio for DGKζ could be indirectly linked to kinase deactivation. A higher M/C ratio indicates high rates of molecular migration from the cytosol to the plasma membrane. Ang II-induced biosynthesis of DAG was implemented using a three-minute pulse, as described in the previous section. The strength of the pulse is described by an arbitrary parameter S_1_. Here, the parameter S_1_ is set at three arbitrary levels: 0.5, 2 and 6. In the absence of a pulse (Figure 3 and Figure S1, solid lines) there is no de novo DAG biosynthesis and the system is fixed in its basal state. In the basal state, both molecules reside in the cytosol with no possibility of translocation.

In the presence of a pulse, the M/C ratios of both PKCα (Figure 3a, dashed line) and DGKζ (Figure 3b, dashed line) increase to their maximum levels followed by gradual clearance of signal. The temporal dynamics depicted in Figure 3 clearly show two phases of translocation. The first is an early phase in which the target and attenuator molecules of DAG signaling migrate from the cytosol to the plasma membrane. The first phase is followed by a second phase where both molecules translocate back to the cytosol. These results indicate that the migration of PKCα and DGKζ from the cytosol to the plasma membrane depends on local DAG concentration at the plasma membrane. It is also linked to the *de novo* biosynthesis rate of DAG, which is controlled throughout the duration. It is also linked to the amplitude of pulse stimulation at the plasma membrane (Figure 3a,b, inset; pulse strength parameter S_1_ = 0.5, 2 and 6).

Translocation of PKCα from the cytosol to the plasma membrane is controlled by two key parameters. The first parameter is DAG concentration at the plasma membrane. Here, the PKCα translocation rate has been set to be directly proportional to DAG concentration (Figure S4 and Table S1). The second parameter is DGK_II_ζ concentration in the cytosol. Here, the PKCα translocation rate is indirectly dependent on DGK_II_ζ concentration in the cytosol, as DGK_II_ζ concentration controls DAG levels at the plasma membrane. In the simulation, when S_1_ is set at low levels [S_1_ = 0.5, 2] a small amount of DAG is generated at the plasma membrane. This induces the migration of a small pool of PKCα to the membrane. PKCα forms complex C_1_ with DGKζ in the membrane compartment and induces the metabolism of DAG to PA. This process restores DAG homeostasis. Once DAG homeostasis is restored, complex C_1_ decomposes into PKC_I_α and DGK_I_ζ. PKC_I_α and DGK_I_ζ, in turn, quickly translocate back to the cytosol without undergoing complete activation and deactivation cycles, respectively. In contrast, during high-intensity stimulation [S_1_ = 6] a large quantity of DAG is generated. This stimulates translocation of a large pool of PKCα from the cytosol to the plasma membrane. High levels of stimulation lead to larger M/C ratios for PKCα (Figure 3a). 

The translocation pattern of DGKζ (Figure 3b, dashed line) is identical to that of PKCα. Upon stimulation, the M/C ratio rapidly increases due to a sharp increase in DAG concentration. Translocation of DGKζ is regulated through the free DAG concentration at the plasma membrane. DGKζ translocation is modeled as a proportional function of free DAG concentration. These results also indicate that high levels of stimulation lead to a larger M/C ratio for DGKζ (Figure 3b). These simulations also show that, for S_1_ = [0.5, 2, and 6], the maximum value of the M/C ratio for PKCα is always larger than the M/C ratio for DGKζ. This is due to the PKCα translocation rate’s dependence on DAG concentration.

### 2.3. Effects of DGKζ Overexpression on the Translocation Characteristics of PKCα in Cardiomyocytes

**Figure 4:**
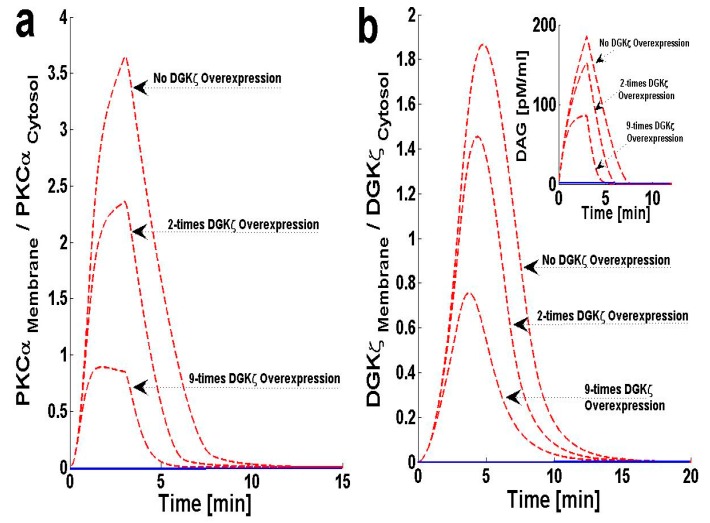
The effect of cardiac-specific overexpression of diacylglycerol kinase ζ (DGKζ) on the membrane-to-cytosol (M/C) ratio of target and attenuator molecules of DAG signaling in cardiomyocytes. For this case, the pulse intensity is set at level 6 for 3 min leading to rapid generation of DAG. The rapid generation of second messenger, in turn, stimulates the translocation of both the DAG target and attenuator molecules from cytosol to membrane. Here, the overexpression of DGKζ is modeled through increasing the ratio of DGKζ to PKCα in the initial conditions. For the no overexpression case, the DGKζ to PKCα ratio is set at 1, whereas for simulations mimicking 2-fold and 9-fold overexpression this ratio is set at 2 and 9 respectively. Here, the solid line represents the non-stimulation condition, whereas the dashed line represents stimulation. (**a**) M/C ratios of PKCα at different overexpression levels of DGKζ. Simulations are performed with no overexpression, 2-fold and 9-fold overexpression of DGKζ molecule. These results show that overexpression of DGKζ molecule restricts the PKCα translocation from cytosol to membrane. At almost 2-fold DGKζ overexpression, a significant reduction in the M/C ratio of PKCα is observed. For 9-fold overexpression, a further reduction in M/C ratio is noticed and the M/C ratio for this case is even lesser than 1. At even higher DGKζ overexpression, the PKCα translocation to membrane is completely eliminated (results not shown). (**b**) M/C ratios of DGKζ at different overexpression levels of DGKζ. The maximum value of M/C ratio first decreases from 1.84 to 1.42 as the overexpression level increase from no overexpression to 2-fold. Interestingly, as the overexpression further increases to 9-fold, the maximum value of M/C ratio decreases to 0.78.

The chance of survival after MI increases upon cardiac-specific overexpression of DGKζ. This is due to the attenuation of post-infarction LV remodeling. The survival rate almost doubles with DGKζ overexpression [14]. Observations in transgenic mice indicate that, independent of hemodynamic effects, DGKζ overexpression attenuates angiotensin II-induced activation of DAG–PKCα signaling and subsequent contractile dysfunction [13,14,15]. Experimental observations show that, in wild type mouse hearts, angiotensin II induces the translocation of PKCα from the plasma membrane to the cytosol [13]. This translocation event is blocked by DGKζ overexpression [13,15]. I set out to address the question of why translocation is blocked by DGKζ overexpression by using a simple regulatory model for local DAG signaling. In this model’s simulations, DGKζ overexpression was implemented by adjusting the DGKζ-to-PKCα ratio under initial conditions. In the case where DGKζ is not overexpressed, the DGKζ-to-PKCα ratio is set at 1. For simulations mimicking 2-fold and 9-fold overexpression over basal levels of DGKζ, the ratios are set at 2 and 9, respectively. Here, the effects of DGKζ overexpression on migration of target and attenuator molecules of DAG signaling (Figure 4a,b) were tracked. Figure 4a shows that overexpression of DGKζ restricts PKCα translocation from the cytosol to the plasma membrane. At 2-fold DGKζ overexpression, a significant reduction in the M/C ratio of PKCα is observed. For 9-fold overexpression, a further reduction in the M/C ratio is observed and the M/C ratio for this case is less than 1. At even higher levels of DGKζ overexpression, PKCα translocation to the plasma membrane can be completely eliminated. These results also show that overexpression of DGKζ only slightly influences the migration characteristics of attenuator molecules of DAG signaling (Figure 4b). These results indicate that the maximum value of the M/C ratio of DGKζ decreases from 1.84 to 0.78 as the overexpression level increases from basal levels to 9-fold overexpression. 

### 2.4. Effects of PKC_I_α^Active^’s Translocation Parameter λ_3_ on DAG–PKCα Signaling Characteristics

**Figure 5:**
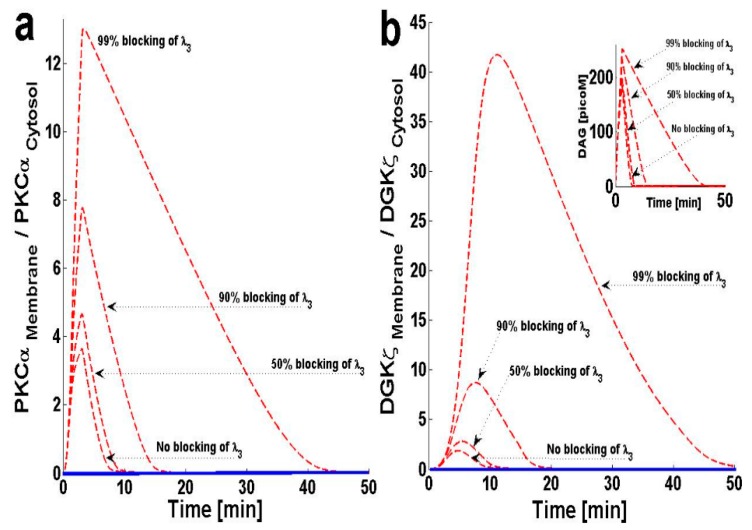
The effect of PKC_I_α^Active^’s re-translocation parameter ‘λ_3_’ on the dynamical signaling characteristics of the DAG–PKCα–DGKζ cascade. For these simulations, the intensity of pulse stimulation is set at 6 with a duration of 3 min, leading to rapid generation of DAG. The rapid generation of second messenger, in turn, stimulates the translocation of both the DAG target and attenuator molecules from cytosol to membrane. Here, the solid line represents the non-stimulation condition, whereas the dashed line represents stimulation. (**a**) M/C ratios of PKCα with respect to the re-translocation parameter of active membrane-bound PKCα enzyme i.e., λ_3_. These results show that as the re-translocation parameter is decreased the maximum value of M/C ratio increases, and the duration for which it is non-negligible is also almost doubled. (**b**) M/C ratios of DGKζ at different inhibition levels of λ_3_. Inhibiting the re-translocation parameter of PKC_I_α^Active^ from membrane to cytosol also increased the M/C ratio of DGKζ, and the duration for which it is non-negligible is prolonged (b-Inset) Temporal dynamics of “DAG” with respect to blocking of parameter λ_3_. These results show that blocking λ_3_ not only increased the maximum level of DAG but also the duration for which it is non-negligible.

In this model, PKCα can cycle between four states: PKC_II_α, PKC_I_α, PKC_I_α^Active^ and PKC_II_α^Active^. PKCα cycling is regulated through processes like translocation, activation, translocation back to the previous location, and deactivation. The model assumes that once PKCα is activated in the membrane compartment, it must translocate back to the cytosol before it is deactivated and enters a dormant state. This assumption is based on several lines of experimental observation describing the activation and deactivation cycles of cPKCs [11,23]. From these experimental results, I propose that PKC_I_α^Active^ translocation back to the cytosol may influence DAG–PKCα signaling. In order to study this question, I blocked the translocation of PKC_I_α^Active^ from the plasma membrane back to the cytosol by decreasing λ_3_. The results indicate that, as λ_3_ is blocked, the maximum value of the M/C ratios for both PKCα and DGKζ increase (Figure 5a,b). 

Figure 5b, inset, shows that blocking this translocation back to the cytosol not only increases the maximum DAG concentration, but also prolongs the duration during which the concentration is non-negligible. These results indicate that inhibition of PKC_I_α^Active^ translocation back to the cytosol reduces the ability of complex C_1_ to metabolize DAG, as PKCα is possibly trapped in the PKC_I_α^Active^ state in the plasma membrane.

### 2.5. Effects of PKC_II_α^Active^’s Deactivation Parameter k_0_ on DAG–PKCα Signaling Characteristics

**Figure 6:**
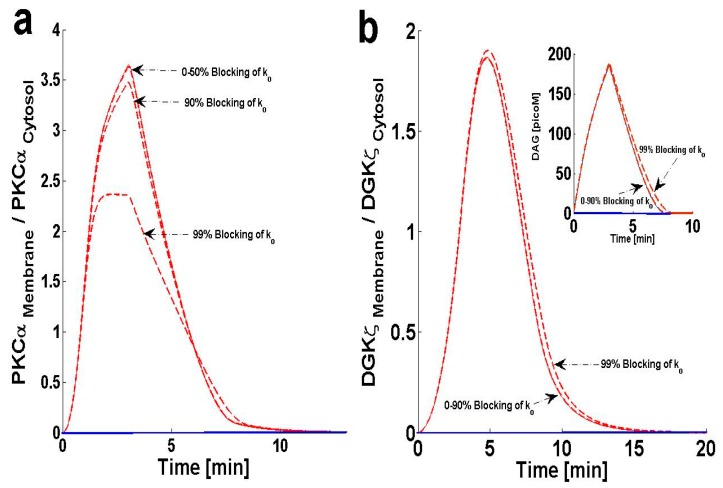
The effect of PKC_II_α^Active^’s deactivation parameter, ‘k_0_’ on the dynamical signaling characteristics of the DAG–PKCα–DGKζ molecular loop. For these simulations, the pulse intensity is set at 6 for 3 min, leading to rapid generation of DAG. The rapid generation of second messenger, in turn, stimulates the translocation of both the DAG target and attenuator molecules from cytosol to membrane. Here, the solid line represents the non-stimulation condition, whereas the dashed line represents stimulation. (**a**) M/C ratios of PKCα at different levels of ‘k_0_’ inhibition. These results show that blocking the deactivation parameter k_0_ effectively reduces the M/C ratio of PKCα, thus restricting the translocation of PKCα from cytosol to membrane. At much higher blocking levels (99% blocking), the M/C ratio decreases to 2.42. This reduction in M/C ratio is due to the disruption in PKCα cycle which is caused by the inhibition of deactivation parameter k_0_. (**b**) M/C ratios of DGKζ at different levels of ‘k_0_’ inhibition. (b, inset) temporal dynamics of “DAG” at different k_0_ inhibition levels. These results show that extensive blocking (0–90%) of the deactivation parameter “k_0_” did not lead to a significant increase in ‘DAG’ levels. Even 99% blocking of the deactivation parameter “k_0_” only leads to a slight increase in the maximum levels of ‘DAG’ and a slight increase in the duration for which it is non-negligible. These results provide an interesting possibility for “k_0_” inhibition as a potential therapeutic target for PKCα in heart failure.

As mentioned in the previous section, the PKCα cycle is regulated through four key processes: translocation, activation, re-translocation and deactivation. Similar to the re-translocation process, the deactivation process can also critically influence the PKCα cycle. Several lines of experimental observation show that, in the cytosol, cPKC is deactivated back to its dormant state through proteins like HSP60. In the next simulation, the effects of the PKC_II_α^Active^ deactivation parameter, k_0_, were tested on DAG–PKCα signaling. The results show that much higher levels of deactivation parameter k_0_ are needed to effectively reduce the M/C ratio of PKCα (Figure 6a). These high levels thus restrict PKCα translocation from the cytosol to the membrane. At very high levels (99% blocking) the maximum M/C ratio decreases to 2.42 (Figure 6a). This reduction in M/C ratio is due to the disruption in the PKCα cycle associated with inhibition of deactivation parameter k_0_. In contrast, the M/C ratio of DGKζ (Figure 6b) slightly increases with k_0_ blocking. The temporal dynamics of DAG are also affected (Figure 6b, inset) at different levels of k_0_ inhibition. These results show that significant blocking (0–90%) of the deactivation parameter “k_0_” does not lead to a significant increase in ‘DAG’ levels. Even 99% blocking of the deactivation parameter “k_0_” only leads to a slight increase in the maximum levels of ‘DAG’ and a slight increase in the duration during which DAG levels are non-negligible. These results point towards the interesting possibility that “k_0_” inhibition may serve as a strategy when designing therapeutic targets related to PKCα during heart failure. 

### 2.6. Effect of Forward Rate Constant, ‘k_2_’, on DAG–PKCα Signaling Characteristics

**Figure 7:**
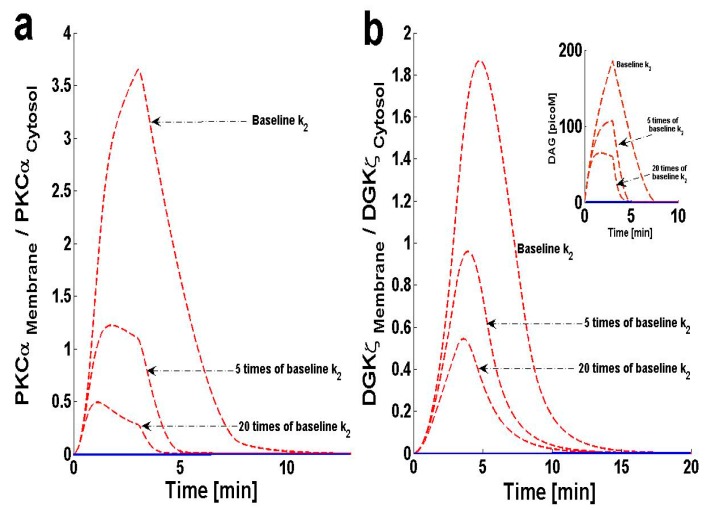
The effect of increasing the forward rate constant, ‘k_2_’ on the dynamical signaling characteristics of the DAG–PKCα–DGKζ signaling complex. The parameter ‘k_2_’ represents the formation rate constant during the interaction of PKC_I_α and DGKζ at plasma membrane to form the biochemical complex C_1_. For these simulations, the pulse intensity is set at 6 for 3 min leading to rapid generation of DAG. The rapid generation of second messenger in turn stimulates the translocation of both the DAG target and attenuator molecules from cytosol to membrane. Here, the solid line represents the basal condition, whereas the dashed line represents stimulation. (**a**) M/C ratios of PKCα at different increasing levels of ‘k_2_. These results show that increasing the formation rate constant k_2_ effectively reduces the M/C ratio of PKCα, and also the duration for which it is non-negligible. (**b**) M/C ratios of DGKζ at different increasing levels of ‘k_2_’. (b-Inset) Temporal dynamics of “DAG” with respect to increasing the parameter ‘k_2_’. These results show that enhancing the parameter “k_2_” aids the formation of complex C_1_ which, in turn, enhances the rate of ‘DAG’ metabolism. These results also reflect that complex C_1_ directly participates in the phosphorylation of ‘DAG’ to ‘PA’ and, therefore, its concentration is critical for regulating the “DAG” homeostasis.

The proposed model suggests that formation of complex C_1_ is crucial for regulating DAG homeostasis at the plasma membrane. Here, I investigate how the kinetics of complex C_1_ formation influence DAG–PKCα signaling. In order to investigate this question, two sets of computational experiments were conducted. The first experimental set involves increasing the forward rate constant (Figure 7), whereas the second set of simulations are focused on blocking the k_2_ parameter (Figure S3), or the rate constant for complex C_1_. The results of this study show that increasing the parameter k_2_ effectively decreases the M/C ratio of PKCα, and also reduces the duration for which the ratio is non-negligible (Figure 7a). These data also show that blocking the parameter k_2_ effectively increases the M/C ratio of PKCα and prolongs the duration for which it is non-negligible (Figure S3a). The results for the M/C ratio of DGKζ (Figure 7b and Figure S3b) also follow the same pattern. 

## 3. Discussion

Dysregulation in cardiac contractile function is a fundamental characteristic of heart failure and has been linked to impaired circulation and fluid homeostasis [24,25]. Chemical agents called positive inotropes, which can enhance cardiac pump function during end-stage heart failure, may provide an attractive therapeutic strategy [24,26,27,28]. Previous work in this field has led to the discovery of agents which can clearly improve contractility in both acute and chronic heart failure [24,26,27,28]. Unfortunately, these agents are associated with high mortality rates, possibly due to desensitization of the entire β-adrenergic system [25,26,27]. Thus, in many clinical settings, the safety of traditional inotropes is controversial [27,28,29,30]. Therefore, there is a critical need for heart failure research to focus on finding therapeutics that are milder alternatives to positive inotropes and do not come with a high risk of β-adrenergic system desensitization [9,10,29,30]. Animal models for heart failure indicate that pharmacological inhibition of PKCα may have therapeutic potential, as agents that regulate PKCα appear to be safer, with milder side effects compared to cAMP elevating agents [8,12,28,29,30,31,32,33,34,35]. The enhanced safety profile of PKCα inhibitors is probably due to their target mechanisms of action. These agents act at the level of sarcoplasmic reticulum and myofilament proteins [9,10,11,12,35]. Unlike traditional inotropic therapies, inotropes that work through inhibition of PKCα are not thought to engage upstream signaling. Thus, treatment with these agents may not result in β-adrenergic system desensitization. Additionally, it remains entirely possible that PKCα inhibition may also affect the heart in ways other than alterations in contractile performance. PKCα inhibition may positively affect ventricular remodeling and the activity of other stress signaling pathways [11,35].

This study proposes PKCα may partially modulate the transition of initial compensatory responses to impaired cardiac function. PKCα-mediated changes and impairments to the calcium cycle and/or causing stiffness patterns in myofilament proteins may regulate the key molecular mechanisms responsible for the transition to contractile dysfunction and development of arrhythmias. Experimental evidence provides a strong causal link between PKCα and impaired ventricular systolic and diastolic functions [8,11,12,35]. Clinical observations show that PKCα inhibition significantly improved cardiac output in heart failure models [8,9,10,11,12,35]. In this study, I investigated the general hypothesis that, in Gαq-induced models of heart failure, PKCα may regulate its own activity through a positive feedback loop. The regulatory model proposed in this work is based on the observation that, during normal cellular conditions (low-intensity stimulation), DGKζ molecules can regulate local PKCα activity by metabolizing its activator DAG in a spatially-selective manner [19,20]. However, during pathological conditions (high-intensity stimulation), local DAG generation may outpace the inhibitory activity of DGKζ, thus activating PKCα. Once active PKCα molecule deactivates DGKζ through phosphorylation, the molecule induces its own activation [19,20]. Based on these observations, I propose a two-compartment regulatory model of local DAG signaling in cardiomyocytes.

The two-compartment model proposed in this study accounts for a sequence of complex events. The model accounts for DAG-induced translocation of PKCα and DGKζ, blocking of PKCα translocation through DGKζ, formation of biochemical complex C_1_ between PKCα and DGKζ, DAG removal through C_1_-catalyzed metabolism, DAG-induced activation of C_1_, and activation of PKCα. In addition, the model accounts for deactivation of DGKζ, re-translocation of active PKCα from membrane to cytosol, eventual deactivation of PKCα in the cytosol to its dormant form, and dephosphorylation and re-translocation of DGKζ back to the cytosol. Through incorporating these molecular details, the proposed model provides a mechanistic understanding of how PKCα and DGKζ molecules may interact within cardiomyocytes during healthy states and during heart failure disease states.

The proposed local regulatory model of DAG signaling in Gαq-induced heart failure is composed of two functional loops. The first is a feedback loop exerting negative influence. The second is a molecular loop exerting positive influence on local DAG signaling. The negative feedback loop is generated due to two signaling events. The first signaling event is the DAG-modulated translocation of PKCα and DGKζ from the cytosol to the plasma membrane. The second signaling event is the formation of a biochemical complex “C_1_” at the plasma membrane. The positive effects on local DAG concentration are generated due to three molecular events. The first is the DAG-modulated activation of complex “C_1_” and its subsequent dissociation into PKC_I_α^Active^ and DGK_I_ζ. The second molecular event is the phosphorylation and deactivation of DGK_I_ζ by PKC_I_α^Active^. The third molecular event is the degradation of PKC_II_α^Active^ from the cytosol. 

DAG is a membrane lipid that has a well-documented role in modulating the activity of numerous proteins, including PKC, nPKC, RasGRPs and receptor channel proteins. Due to its broad range of effects, DAG signaling is necessary for normal cellular function. However, DAG signaling must be exquisitely regulated [36,37]. Observations indicate that persistent DAG accumulation may induce malignant cellular transformations and therefore any dysregulation in the molecular apparatus which regulates its metabolism may result in a disease state [36]. A multitude of detrimental effects such as alterations in insulin signaling, apoptosis, endoplasmic reticulum stress and mitochondrial dysfunction have been linked to persistent intracellular lipid accumulation [38,39,40,41,42,43,44,45]. As the heart requires a lot of energy for proper function, lipids serve as a source of this energy [37]. However, persistent accumulation of lipids in the heart can lead to physiological dysfunction and death [37]. DAG accumulation has been linked to cardiac dysfunction [39,44]. The exact cause of lipid toxicity, however, is not clear. It is believed that accumulation of lipids disrupts β-AR signaling through PKC activation, thus altering normal physiological responses to stress [37]. This study assumes that, during normal physiological function, DAG homeostasis at the plasma membrane is regulated through a negative feedback signal (Figure 1; Green color pulse and signaling loop). The balance between local DAG biosynthesis and its removal is probably disrupted during this disease state. The disruption in DAG homeostasis is due to a positive feedback signal (Figure 1; Red color pulse and signaling loop). During normal cellular functions, the amount of DAG generated at the local plasma membrane site is quickly removed through C_1_-mediated metabolism of local DAG. In the pathological state, however, local rates of DAG biosynthesis may far exceed the C_1_-mediated removal rate, leading to a net accumulation of DAG at specific locations in the plasma membrane compartment. These accumulations then activate and destabilize the complex C_1_. Once active, C_1_^A^ dissociates into PKC_I_α^Active^ and DGK_I_ζ. The PKC_I_α^Active^ molecule further enhances the positive feedback effect by phosphorylating and deactivating DGK_I_ζ. According to the proposed model, when local DAG levels and DAG biosynthesis rates are low, a negative feedback loop is induced to facilitate DAG removal from local sites. In contrast, a higher local DAG biosynthesis rate leads to a positive feedback signal. The structure of the proposed model suggests that, during the disease state, DAG may lead to its own persistent accumulation through a positive feedback signaling loop.

This study also explains that, during normal physiological function, the precise spatiotemporal regulation of “DAG” homeostasis may not principally depend on increased activity of its effector molecules PKCα and DGKζ. Instead, the proposed model suggests that DAG homeostasis is maintained through agonist-dependent, site-specific migration of effector molecules from the cytosol to the plasma membrane. Accordingly, DAG signaling capacity at the plasma membrane is critically dependent on the migration characteristics of effector molecules (DAG-dependent) to the plasma membrane and the localization, anchoring, and activation state of these effectors at the plasma membrane. The model proposes a delicate balance between the membrane translocation of effector molecules and their return to the cytosol that directly influences the amplitude and duration of DAG signaling at the plasma membrane. The results presented here indicate the possibility of a DAG-modulated functional coupling between PKCα and DGKζ. These simulated results are based on experimental observations made in cardiomyocytes. The Ang-II-induced membrane translocation of PKCα has been observed [11,12,13,14,15]. At least one previous experimental study [12] indicates that inhibiting PKCα translocation in G_αq_-induced systolic and diastolic dysfunction may provide a mild therapeutic advantage during heart failure. Furthermore, experimental observations indicate enhanced DGKζ expression in infracted hearts [46]. These results reflect that agonist-mediated translocation of PKCα and DGKζ is modulated by DAG concentration at the plasma membrane. This simulation mimics these translocation events by modeling them through simple kinetic steps (Materials and Methods, Equations (2) and (4)). The rate constants of these kinetic steps are described by a simple proportional function of DAG concentration (Supplementary Material 1; Figure S4 and Supplementary Material 2; Table S1). This ad-hoc functional arrangement was developed for the sake of simplicity. The assumptions behind this mathematical expression are a simplified model of translocation events, and it may be argued that the simplifying assumptions render the model unrealistic. However, computational modeling requires accounting for complex molecular details involved using simplifying assumptions. Some of these complex molecular details are Ca^2+^ /DAG release and diffusion, DAG and Ca^2+^ binding to certain domains, the possible interaction of PKCα and DGKζ with translocating rails and scaffolding proteins [46], formation of macromolecular complexes [19,20], and precise anchoring at specific sites/domains. Modeling all these complex molecular details is beyond the scope of this study. The model proposed here is a representation of PKCα and DGKζ translocation that may neglect many important molecular details, but it was chosen to elucidate the influence of DAG concentration on PKCα and DGKζ migration from the cytosol to the plasma membrane through a simple mathematical expression. 

As mentioned above, the membrane translocation events of PKCα and DGKζ in this study are described through first order kinetic functions (Table S1). The rate constants of these functions were obtained by fitting the translocation kinetics [13,20,45,46]. Interestingly, experimental evidence supports a membrane-directed PKCα translocation in Gαq-induced heart failure^13^. Evidence, also shows prolonged elevation of DGKζ in post MI hearts [46]. Additionally, data from T cells is also used within this study to further validate the description of PKCα and DGKζ translocations from the cytosol to the cell membrane [20,45]. Interestingly, MI is followed by an inflammatory reaction, and observations from previous study suggest that elevated DGKζ expression in the infracted area is due to infiltrating macrophages and granulocytes [46]. One limitation of the first order description of PKCα translocation presented here is its dependence on DAG rather than on calcium Ca^+2^. PKCα translocation is mainly driven by calcium binding; the C_2_ domain is the main stimulating factor for enzyme migration. However, this assumption was made because DAG and IP_3_ generation precedes Ca^+2^ release. In a PKC-independent manner, DAG generation stimulates extracellular Ca^+2^ entry via the transient receptor potential canonical 3 (TRPC_3_) channel in sarcolemma [47]. This Ca^+2^ release is independent of intracellular calcium stores [47]. Thus, the assumption relating to DAG-dependent translocation may have some physiological basis. The assumption exists within the proposed model mainly for the purpose of simplification. 

The biological function of the PKCα molecule in the proposed model is regulated through a four-step cycle [11,22]. The four steps within this model are translocation, activation, redistribution/re-translocation and deactivation. The model assumes that inactive but catalytically competent PKC_II_α is stored in the cytosol. *De novo* synthesis generates a rather unstable naïve form of PKC_II_α which constitutively undergoes a sequence of ordered priming and autophosphorylations. This sequence produces a mature, inactive, phosphatase-resistant, and proteasome-resistant molecule [22]. These phosphorylations are essential for PKCα stability and catalytic competence [22]. The PKC life cycle is complex and modulated through precise and tightly-coupled molecular events [22]. Evidence suggests that, before PKC become responsive to second messengers, it must first be phosphorylated at three conserved positions [22]: Thr500, Thr641 and Ser660. This model does not attempt to model *de novo* synthesis or the subsequent processes of enzyme maturation through phosphorylation. This study assumes the cytosol contains sufficient amounts of mature, second-messenger-responsive PKC_II_α enzyme. In these simulations, this is modeled by setting the initial conditions such that only the concentration of PKC_II_α is non-negligible. The concentration of all other forms of PKCα is initially set at negligibly small values.

Here, the time scales of results presented from in silico experiments (Figure 3, Figure 4, Figure 5, Figure 6 and Figure 7) are within 20–50 min, which seems to be in contrast to much longer time scales of experimental data (3–21 days) [13,46]. However, this model was validated against data from animal models [13,46] on the scale of days and weeks as shown in Supplementary Materials (Figures S5–S8). Thus, this model shows that though the setting of heart failure is complex, a simplified model could be used to provide some mechanistic basis. Here, we are not showing the heart failure time scales of years (in humans) however, our model is able to generate activation and translocation data even at that time scales. This work also shows that overexpression of DGKζ can reduce DAG-induced PKCα activation (Figure 4) [13,14,15]. Angiotensin II-induced local DAG generation stimulates the translocation of PKCα from the cytosol to the cellular membrane. As explained above, this translocation event is modeled through a simple proportionality function of DAG concentration. However, experimental observations also show that DGKζ overexpression restricts PKCα migration from the cytosol to the membrane compartment [13,14,15]. Here, the inverse phenomenon is modeled through C_1_-mediated metabolism of DAG. Since the translocation rate of PKCα, λ_3,_ is directly proportional to DAG concentration, any reduction in DAG concentration at the plasma membrane will reduce PKCα translocation to the plasma membrane. This simplistic description of complex interactions between DGKζ and PKCα molecules was chosen for simplicity. As overexpression of DGKζ is controlled in silico, it produces a dose–response curve showing dose-dependent inhibition of PKCα translocation (Figure 4a). These results are consistent with previous experimental observations linking DGKζ overexpression to reduction in PKCα translocation in cardiomyocytes [13]. These results also explain why survival after MI is improved if cardiac-specific overexpression of DGKζ is present [14]. The survival rate almost doubles with DGKζ overexpression.

The results of this study indicate a role for PKC_I_α^Active^ re-translocation to the cytosol (Figure 5). According to the model, once PKCα is active, it is not available to form complex C_1_ and participate in DAG degradation. PKC_I_α^Active^ re-translocation is necessary for the PKCα cycle to function and necessary for complex C_1_ formation. The results of this study show that blocking the re-translocation process increases DAG levels (Figure 5). This model shows that dose-dependent blocking of re-translocation leads increased DAG in the membrane compartment (Figure 5b, Inset). These results explain the role of PKCα shuttling in maintaining DAG homeostasis.

While this model is based upon experimental results, its assumptions, as well as its consequences, require further testing. One of the key assumptions in the proposed translocation-dependent model is that local DAG generation is stimulated through agents such as angiotensin II or phenylephrine. This assumption is supported by observation that, in cardiomyocytes, Ang II and phenylephrine induced the local generation of DAG and subsequent translocation of PKCα from the cytosol to the plasma membrane [7,13,14,15,48,49,50]. Interestingly, other stimulating agents have only a small or negligible effect on PKCα translocation in cardiomyocytes [13]. This indicates that cPKC translocation in cardiomyocytes is highly dependent on the stimulating pathways and is consistent with the assumption that local DAG generation in response to angiotensin II-mediated activation of GPCR pathway is regulated through a complex process [48]. This model does not attempt to model this complex process. The model uses stimulating variable i.e., parameter S_1_ to mimic the GPCR agonist angiotensin II. A brief pulse of parameter S_1_ at varying intensity represents the influence of angiotensin II on local DAG biosynthesis [48]. This simple approach ignores the complex details of angiotensin II-induced DAG biosynthesis [48]. Furthermore, the stimulation pulse was only applied for a 3-minute duration (Figure 3, Figure 4, Figure 5, Figure 6 and Figure 7 and Figures S1–S3), that is why both PKCα and DGKζ return to basal levels after 10 min. This is in contrast to previous observations, showing significant PKCα elevation in angiotensin II-treated WT mice for 14 days [13]. This difference is due to the duration of pulse stimulation as shown in a Figure S5, where pulse is applied for 30 min leading to persistence of DAG and PKCα. In conclusion, a two-compartment model was developed for regulating DAG homeostasis in Ang II-induced heart failure. This computational model is a promising tool to study mechanisms of DAG regulation in the context of heart failure. This model may be used to identify novel therapeutic targets with the aim of improving survival and quality of life outcomes in heart failure patients. 

## 4. Materials and Methods

### 4.1. Biochemical Reactions

The biochemical interactions described in this study include Gαq-induced local DAG–PKCα–DGKζ signaling in cardiomyocytes [12,13,14,15,16]. The computationally-modeled interactions of this local molecular loop (Figure 1 and Figure 2) are based on standard Michaelis–Menten kinetics. The following sets of biochemical reactions are used to describe the molecular interactions of other molecules with this loop. The dynamic variables used are: (1) DAG, representing second messenger diacylglycerol, (2) DGKζ representing diacylglycerol kinase, (3) PKCα, representing the α isoform of protein kinase C. A subscript I represents the concentration in the first compartment, which is the plasma membrane. Subscript II denotes the concentration in the second compartment, which is the cytosol. Superscript A represents the activated form of a molecule. Subscript P represents the phosphorylated form of a molecule. The phosphatase P is approximated as a fixed parameter. The parameter S_1_ denotes AngII-induced stimulation, leading to the rapid generation of DAG.

(1) $$\begin{array}{l} S_{1}\overset{k_{1}}{\to }\;\mathrm{DAG}\; \end{array}$$

(2) $$\begin{array}{l} {\mathrm{PKC}}_{\mathrm{II}}\alpha \overset{\lambda_{0}=\;f(\mathrm{DAG})}{\to }{\;\mathrm{PKC}}_{I}\alpha \end{array}$$

(3) $$\begin{array}{l} {\mathrm{PKC}}_{I}\alpha \overset{\lambda_{00}}{\to }{\;\mathrm{PKC}}_{\mathrm{II}}\alpha \end{array}$$

(4) $$\begin{array}{l} {\mathrm{DGK}}_{\mathrm{II}}\zeta \overset{\lambda_{5}=\;f\;(\mathrm{DAG})}{\to }{\;\mathrm{DGK}}_{I}\zeta \end{array}$$

(5) $$\begin{array}{l} {\mathrm{DGK}}_{I}\zeta \overset{\lambda_{55}}{\to }{\;\mathrm{DGK}}_{\mathrm{II}}\zeta \end{array}$$

(6) $${\mathrm{DGK}}_{I}\zeta +{\;\mathrm{PKC}}_{I}\alpha \overset{k_{2}}{\underset{k_{3}}{⇄}}{\;C}_{1}$$

(7) $$C_{1}+\;\mathrm{DAG}\;\overset{k_{4}}{\underset{k_{5}}{⇄}}C_{1}{}^{A}$$

(8) $$\begin{array}{l} C_{1}{}^{A}\overset{k_{6}}{\to }{\;\mathrm{PKC}}_{I}\alpha^{A}+{\;\mathrm{DGK}}_{I}\zeta \end{array}$$

(9) $$\begin{array}{l} {\mathrm{PKC}}_{I}\alpha^{A}\overset{\lambda_{3}}{\to }{\;\mathrm{PKC}}_{\mathrm{II}}\alpha^{A} \end{array}$$

(10) $$\begin{array}{l} {\mathrm{PKC}}_{\mathrm{II}}\alpha^{A}\overset{\lambda_{4}}{\to }\;[\;] \end{array}$$

(11) $$\begin{array}{l} {\mathrm{PKC}}_{\mathrm{II}}\alpha^{A}\overset{k_{0}}{\to }{\;\mathrm{PKC}}_{\mathrm{II}}\alpha \end{array}$$

(12) $${\mathrm{DGK}}_{I}\zeta +{\;[\mathrm{PKC}}_{I}\alpha {]}^{A}\overset{k_{7}}{\underset{k_{8}}{⇄}}C_{2}\overset{k_{9}}{\to }{\;[\mathrm{PKC}}_{I}\alpha {]}^{A}+{\;\mathrm{DGK}}_{I}\zeta_{P}$$

(13) $$C_{1}+DAG\overset{k_{10}}{\underset{k_{11}}{⇄}}C_{3}\overset{k_{12}}{\to }C_{1}+{\;\mathrm{DAG}}_{P}$$

(14) $$\begin{array}{l} DAG_{P}+\;P\;\overset{k_{13}}{\to }\;\mathrm{DAG}\;+\;P \end{array}$$

(15) $$\begin{array}{l} DAG_{P}+\;P\;\overset{k14}{\to }\;P.A \end{array}$$

(16) $$\begin{array}{l} {\mathrm{DGK}}_{I}\zeta_{P}+\;P\;\overset{k_{15}}{\to }{\;\mathrm{DGK}}_{I}\zeta +\;P \end{array}$$

Signaling in this loop is initiated by local generation of DAG at the lipid membrane in angiotensin-stimulated cardiomyocytes. This generation of the DAG second messenger is described through Equation (1). DAG generation stimulates the migration of dormant and inactive PKC_II_α from the cytosol to the lipid membrane. PKCα translocation is described in Equation (2). Here, the migration rate λ_0_ is described through a function which is directly proportional to DAG concentration (Figure S4 and Table S1). PKC_I_α is now an inactive α-molecule in the lipid membrane compartment. PKC_I_α then translocates back to the cytosol with a fixed migration rate λ_00_, described in Equation (3). Here, DGK_II_ζ, which attenuates DAG signaling, also migrates from the cytosol to the plasma membrane in a DAG-dependent manner. This migration event is described in Equation (4). DGK_I_ζ, the ζ molecule at the plasma membrane, can also translocate back to the cytosol, as described in Equation (5). Once at the plasma membrane, both PKC_I_α and DGK_I_ζ bind one another, forming a biochemical complex C_1_ [19,20] This event is described in Equation (6). Under basal conditions, complex C_1_ regulates local levels of DAG by metabolizing the second messenger through phosphorylation. These regulatory steps are modeled in Equations ((13)–(15)). However, upon stimulation, local DAG levels rise very sharply. These levels outpace the ability of complex C_1_ to convert DAG to PA. The net accumulation of DAG activates complex C_1_ through PKC_I_α binding within C_1_ [10,19,20]. This activation event is described in Equation (7). The active form C_1_^A^ is unstable and quickly dissociates into PKC_I_α^Active^ and DGK_I_ζ, as modeled in Equation [19,20] (8). In turn, PKC_I_α^Active^ deactivates DGK_I_ζ through phosphorylation [21]. This deactivation event is described in Equation (12). Through this event, PKCα prolongs its own activation. The active form of PKC_I_α^Active^ α-isoform migrates from the plasma membrane to the cytosol [11] as described in Equation (9). Here, the migration rate λ_3_ is set as a fixed parameter. Once inside the cytosol, the active form of PKC_II_α^Active^ can either follow a degradation pathway as described in Equation (10) or undergo deactivation to its dormant form [11,22]. The fate of PKC_II_α^Active^ is modeled in Equation (11). DGK_I_ζ_P_ dephosphorylation is described by Equation (16).

### 4.2. Induction

During simulations, the angiotensin II-mediated activation of the GPCR pathway [7] is mimicked through a brief 3-minute pulse which leads to local biosynthesis of second messenger DAG in the membrane compartment, thus inducing the translocation and activation of DAG effector molecules.

### 4.3. Temporal Dynamics

The differential equations (Supplementary Material 3: Equations (17)–(28) resulting from the above interactions (Equations (1)–(16)) were integrated through nonlinear solvers using MATLAB (MathWorks). The dynamical coefficients’ values were estimated from limited experimental data [11,12,13,14,15,16,17,18,19,20,21,22,23,46,47,48,49,50]. Unless otherwise stated, all of the molecular concentrations in the model are expressed as pg/ml and time is represented in seconds. 

## Supplementary Materials

Supplementary materials can be found at https://www.mdpi.com/1422-0067/21/7/2561/s1.
