# Supplementary material for: Increase in PKCα Activity during Heart Failure Despite the Stimulation of PKCα Braking Mechanism
Source: Int J Mol Sci. 2020 Apr 7;21(7):2561. doi: 10.3390/ijms21072561 (PMC7177253; doi:10.3390/ijms21072561)
Supplement: Supplementary file 1 [file ijms-21-02561-s001.pdf]

Supplementary 1: Supplementary Figures

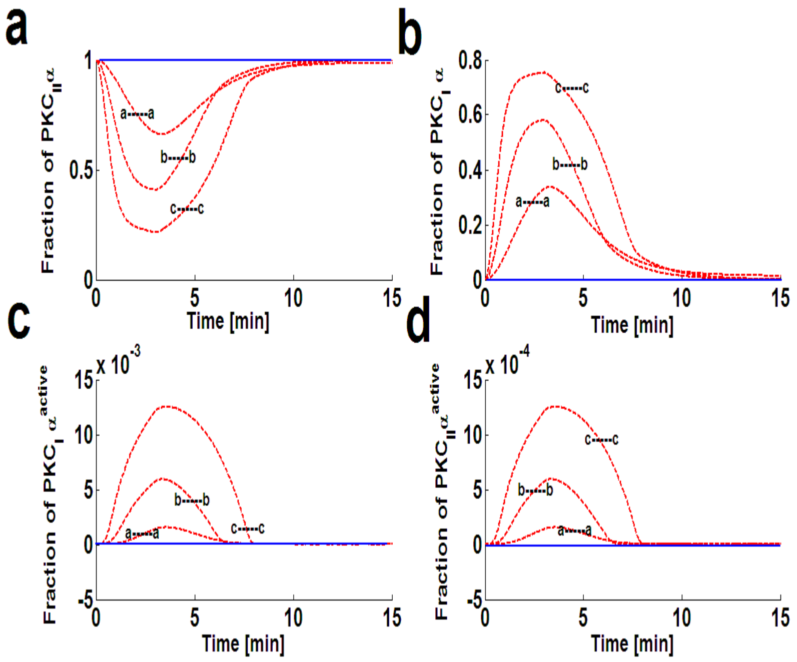

Figure S1. Dynamical characteristics of all four isoforms of PKCα. These temporal dynamics corresponds to results presented in figure 3 (relative distribution of PKCα and DGKζ in membrane and cytosol compartments in response to Ang-II like stimulation). These results show that in the basal conditions all the PKCα resides in the dormant form in cytosol i.e., PKCIIα. However, on stimulation the enzyme is distributed into all four forms i.e., PKCIIα, PKCIα, PKCIIα^{Active} and PKCIα^{Active}. The extent and duration of this distribution is directly dependent on the Ang-II like stimulation and hence, on DAG. Here, three different levels of stimulation are used (Figure 3). The symbol a—a represents a pulse strength of 0.5, b—b represents a pulse strength of 2.0, and c—c represents the pulse strength of 6.0. Higher levels of pulse (c—c) correspond to higher levels of DAG generation. Here, the solid line represents the basal condition, whereas the dashed line represents stimulation. (a) Fraction of inactive and dormant α-enzyme in cytosol i.e., PKCIIα. For the case of low stimulation levels (a—a, b—b), only a small drop from unity is observed. However, for higher stimulation levels (c—c), the drop is much more prominent and takes longer time to recover. This difference is due to much higher levels of PKCα translocation from cytosol to membrane in the case of higher stimulation intensity. (b) Fraction of inactive α-enzyme in plasma membrane i.e., PKCIα. In the basal conditions there is no change in the PKCIα fraction. However, during stimulation (a—a, ---- c—c) the PKCIα fraction quickly increases followed by a resolution phase. The maximum levels of PKCIα and duration for which it is non-negligible is dependent on the stimulation strength. (c) Fraction of active α-enzyme in plasma membrane i.e., PKCIα^{Active}. During non-stimulation condition no change in the fraction of PKCIα^{Active} is observed. However, on stimulation the PKCIα^{Active} levels quickly increase to a maximum (induction phase) followed by a gradual resolution to basal levels. Again the maximum levels of PKCIα^{Active} and duration for which it is non-negligible is dependent on the stimulation strength. (d) Fraction of active α-enzyme in cytosol i.e., PKCIIα^{Active}. During the basal condition there is no change in the fraction of PKCIIα^{Active}. Due to fast deactivation rate of PKCIIα^{Active}, very little change is observed in the fraction of PKCIIα^{Active} even in the case of stimulation.

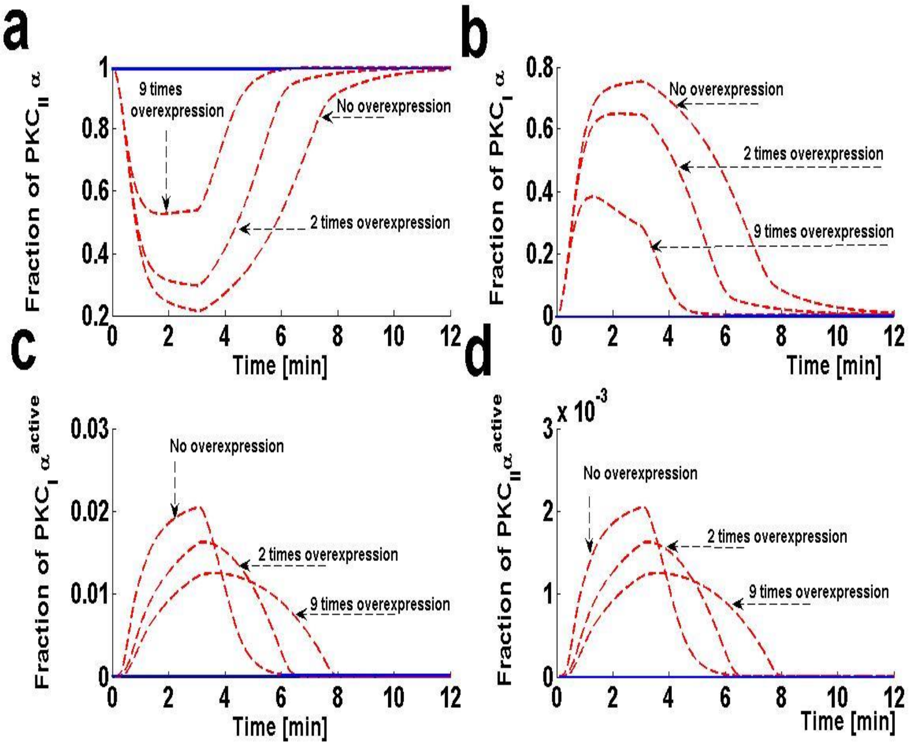

Figure S2. The effect of DGKζ overexpression on the dynamical characteristics of different forms of PKCα enzyme and second messenger DAG. These temporal dynamics corresponds to results presented in figure 4 (relative distribution of PKCα and DGKζ in membrane and cytosol compartments in response to the DGKζ overexpression at the pulse strength of 6.0). Here, the solid line represents the basal condition, whereas the dashed line represents stimulation. These results show that for basal conditions there is no change in the distribution patterns of PKCα enzyme and all the PKCα resides in its dormant form i.e., $PKC_{II}\alpha$. In contrast, during stimulation the PKCα enzyme actively translocate from cytosol to membrane. This translocation event is directly regulated by second messenger DAG. The extent and duration of this distribution is dependent on the levels of pulse like stimulation at plasma membrane. (a) Temporal dynamics of the inactive and dormant α-enzyme in cytosol i.e., $PKC_{II}\alpha$. For the case of no DGKζ overexpression the $PKC_{II}\alpha$ fraction quickly drops from unity to almost 0.2 followed by slow recovery in almost 12 minutes. In the case of DGKζ overexpression the drop in the fraction of $PKC_{II}\alpha$ is reduced. 2 and 9 times overexpression leads to a drop from unity to 0.35 and 0.58 respectively, followed by a rapid recovery. (b) Temporal dynamics of $PKC_{I}\alpha$ fraction. For the case of no DGKζ overexpression the $PKC_{I}\alpha$ fraction quickly increases to 0.8 followed by clearance to basal levels in almost 12 minutes. In case of DGKζ overexpression (2 and 9 times) the maximum induction levels of $PKC_{I}\alpha$ fraction are reduced and also the resolution to basal levels is faster. (c) Temporal dynamics of active α-enzyme's fraction in membrane i.e., $PKC_{I}\alpha^{Active}$. (d) Temporal dynamics of active α-enzyme's fraction in cytosol i.e., $PKC_{II}\alpha^{Active}$.

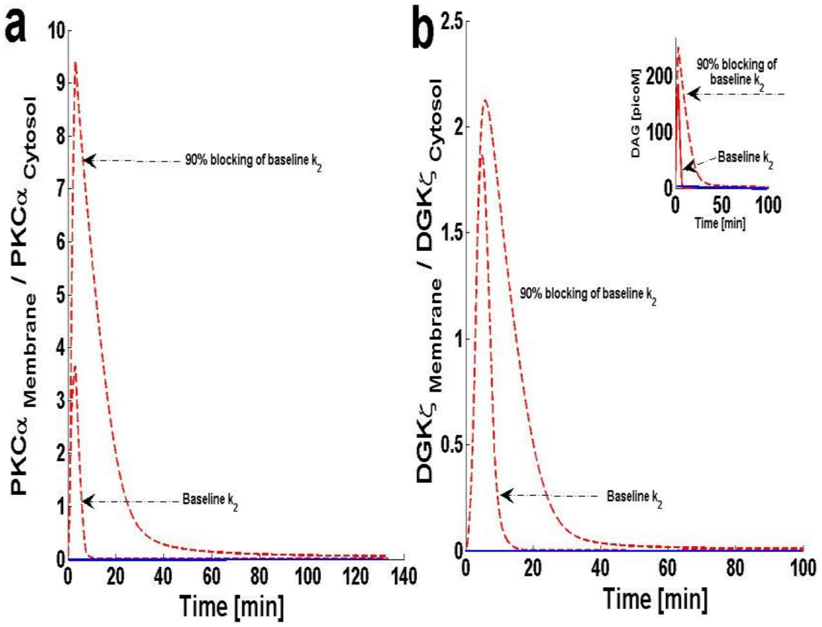

Figure S3. The effect of decreasing the forward rate constant, 'k_2' on the dynamical signaling characteristics of DAG-PKCα-DGKζ signaling complex. The parameter 'k_2' represents the formation rate constant during the interaction of PKC$_1\alpha$ and DGKζ at plasma membrane to form the biochemical complex C_1. For these simulations the pulse intensity is set at 6 for three minutes leading to rapid generation of DAG. The rapid generation of second messenger, in turn, stimulates the translocation of both the DAG target and attenuator molecules from cytosol to membrane. Here, the solid line represents the basal condition, whereas the dashed line represents stimulation. (a) M:C ratio of PKCα at different increasing levels of 'k_2'. These results show that decreasing the formation rate constant k_2 effectively increases the M:C ratio of PKCα, and also the duration for which it is non-negligible. (b) M:C ratio of DGKζ at different levels of 'k_2'. (b-inset) Temporal dynamics of "DAG" with respect to decreasing the parameter 'k_2'. These results show that decreasing the parameter "k_2" reduces the formation of complex C_1 which, in turn, reduces the rate of 'DAG' metabolism. These results also reflect that complex C_1 directly participates in the phosphorylation of 'DAG' to 'PA' and therefore, its concentration is critical for regulating the "DAG" homeostasis.

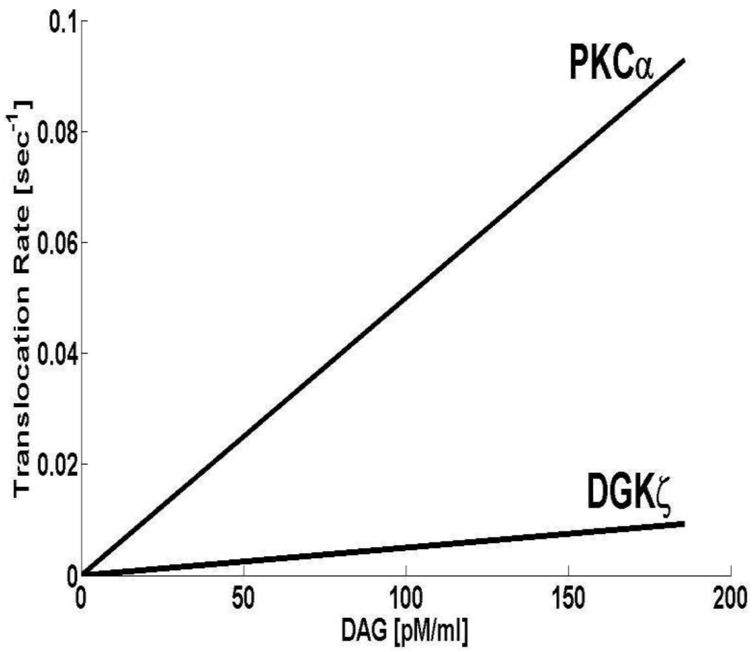

Figure S4. The translocation rates of PKCα and DGKζ molecules described as a linear proportional function of DAG concentration. The translocation of PKCα and DGKζ molecules from cytosol to membrane is described through simple kinetic steps (**Materials and Methods Eqs. 2 &4**). The rate constant of these kinetic steps i.e., λ_0 & λ_5 are described as simple proportional function of DAG concentration.

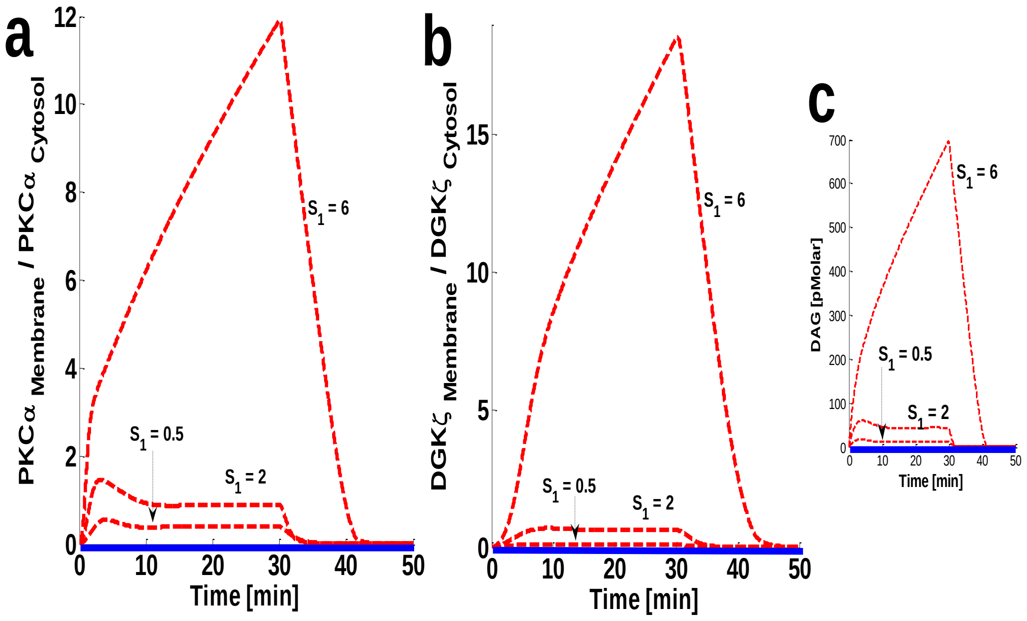

Figure S5. The effect of prolonged duration of stimulation on Membrane to cytosol (M/C) ratio of target & attenuator molecules of DAG signaling. These results show that prolonged pulse like (duration of, 30 minutes and three different levels of pulse strength are used i.e., "S_1" = 0.5,2 & 6) stimulation leads to the rapid generation of DAG and persistence. The generation of second messenger, in turn, stimulates the translocation of both the target and attenuator molecules of DAG signaling from cytosol to membrane. Here, the solid line represents the non-stimulation condition, whereas the dashed line represents stimulation. (a) M/C ratio of PKCα with respect to different levels of pulse like stimulation mimicking the GPCR agonist angiotensin II (Ang. II). These results show that Ang. II like stimulation leads to rapid de-novo generation of DAG, which, in turn, stimulates the

translocation of both PKCα and DGKζ from cytosol to membrane. Here, the translocation rates are set as linear functions of DAG concentration. At low stimulation levels only small amount of DAG is generated at plasma membrane thus, inducing the migration of only a small pool of PKCα to membrane. At membrane PKCα forms a biochemical complex C_1 with DGKζ and stimulates the DAG conversion to PA. Once DAG homeostasis is restored the complex C_1 decomposes into DGK$_1\zeta$ and PKC$_1\alpha$ molecules; these, in turn, quickly re-translocate to cytosol compartment. At higher stimulation levels much larger quantity of DAG is generated, thus stimulating the translocation of much larger pool of PKCα from cytosol to membrane. High intensity stimulation leads to much larger M/C ratio of PKCα and enhanced residence time in membrane compartment. (b) M/C ratio of DGKζ with respect to different levels of stimulation. The DGKζ M/C ratio rapidly increases due to a sharp increase in DAG concentration on stimulation. Here, the translocation event of DGKζ is also modeled as a linear proportional relationship to the free concentration of DAG. (c) DAG concentration in plasma membrane in response to a 30-minute pulse stimulation at plasma membrane. ("S_1" = 0.5,2 & 6).

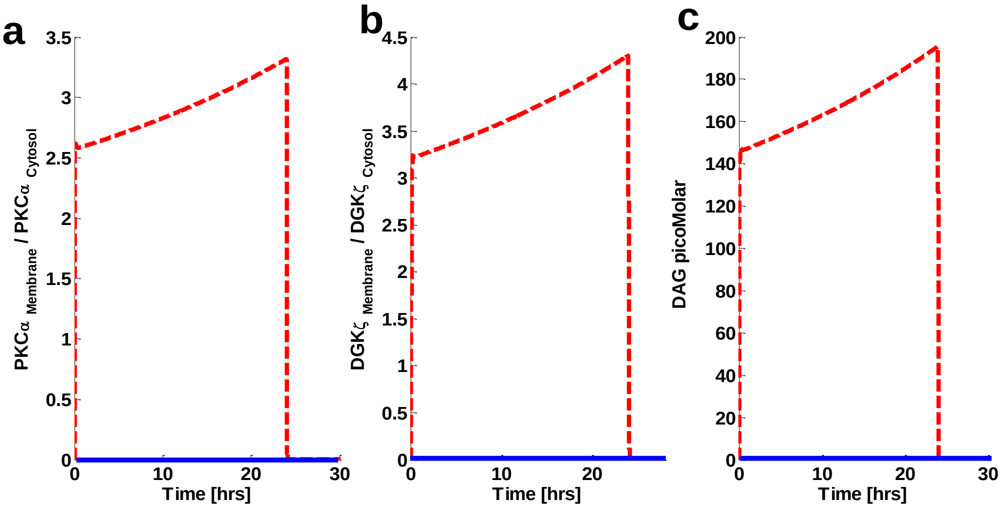

Figure S6. The effect of prolonged duration of stimulation on Membrane to cytosol (M/C) ratio of target & attenuator molecules of DAG signaling. These results show that prolonged pulse like (duration of, 24 hours) stimulation leads to the rapid generation of DAG and persistence. The generation of second messenger, in turn, stimulates the translocation of both the target and attenuator molecules of DAG signaling from cytosol to membrane. Here, the solid line represents the non-stimulation condition, whereas the dashed line represents stimulation. (a) M/C ratio of PKCα with respect to pulse like stimulation mimicking the GPCR agonist angiotensin II (Ang. II). These results show that Ang. II like stimulation leads to rapid de-novo generation of DAG, which, in turn, stimulates the translocation of PKCα from cytosol to membrane. (b) M/C ratio of DGKζ. The M/C ratio rapidly increases due to a sharp increase in DAG concentration on stimulation. (c) DAG concentration in plasma membrane in response to a 24hour pulse stimulation at plasma membrane.

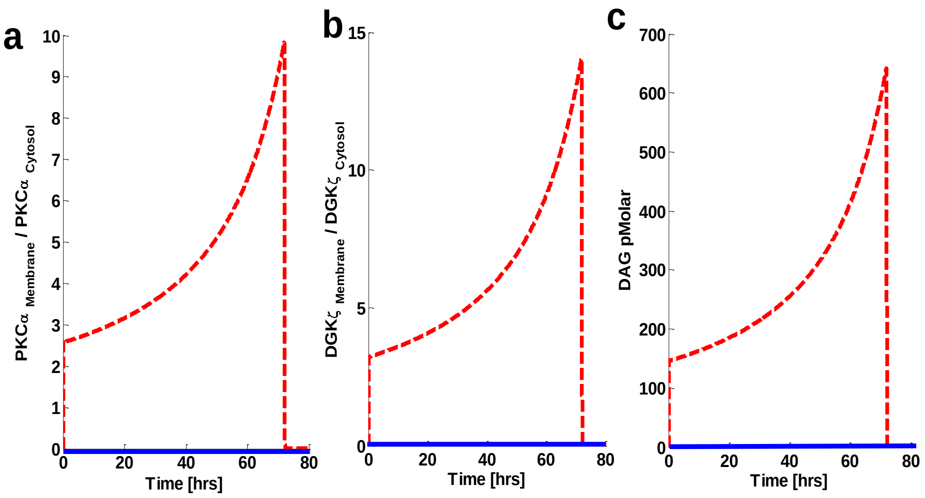

Figure S7. The effect of prolonged duration of stimulation on Membrane to cytosol (M/C) ratio of target & attenuator molecules of DAG signaling. These results show that prolonged pulse like (duration of, 3 days) stimulation leads to the rapid generation of DAG and persistence. The generation of second messenger, in turn, stimulates the translocation of both the target and attenuator molecules of DAG signaling from cytosol to membrane. Here, the solid line represents the non-stimulation condition, whereas the dashed line represents stimulation. (a) M/C ratio of PKCα with respect to pulse like stimulation mimicking the GPCR agonist angiotensin II (Ang. II). These results show that Ang. II like stimulation leads to rapid de-novo generation of DAG, which, in turn, stimulates the translocation of PKCα from cytosol to membrane. (b) M/C ratio of DGKζ. The M/C ratio rapidly increases due to a sharp increase in DAG concentration on stimulation. (c) DAG concentration in plasma membrane in response to a 72 hour pulse stimulation at plasma membrane.

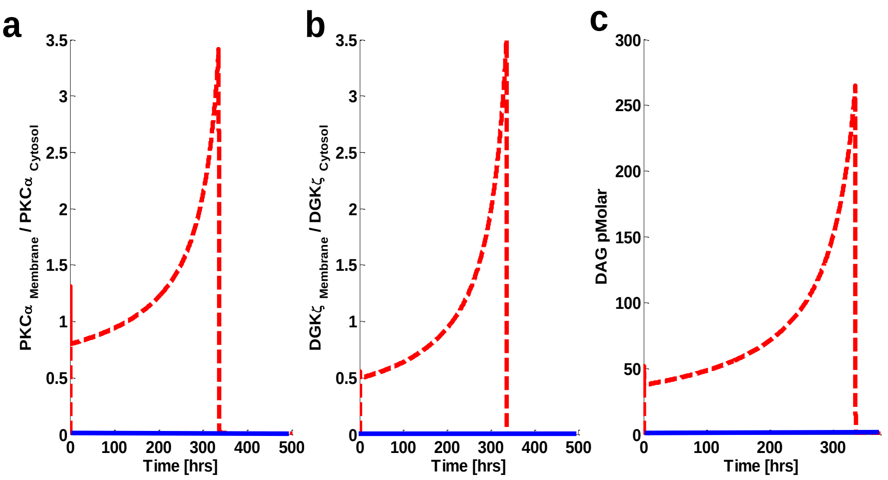

Figure S8. The effect of prolonged duration of stimulation on Membrane to cytosol (M/C) ratio of target & attenuator molecules of DAG signaling. These results show that prolonged pulse like (duration of, 14 days) stimulation leads to the rapid generation of DAG and persistence. The generation of second messenger, in turn, stimulates the translocation of both the target and attenuator molecules of DAG signaling from cytosol to membrane. Here, the solid line represents the non-stimulation condition, whereas the dashed line represents stimulation. (a) M/C ratio of PKCα with respect to pulse like stimulation mimicking the GPCR agonist angiotensin II (Ang. II). These results show that Ang. II like stimulation leads to rapid de-novo generation of DAG, which, in turn, stimulates the translocation of PKCα from cytosol to membrane. (b) M/C ratio of DGKζ. The M/C ratio rapidly increases due to a sharp increase in DAG concentration on stimulation. (c) DAG concentration in plasma membrane in response to a 14 days pulse stimulation at plasma membrane.

Supplementary Material 2: Table S_1 Numerical values of biochemical rate parameters for the Gαq-induced local DAG-PKCα-DGKζ signaling as described in Materials and Methods Equations1-16.

**Parameter**	**Description**	**Numerical Values**
k_1	Kinetic rate constant for DAG generation.	0.25 sec^{-1}
k_2	Association rate constant $DGK_I\zeta$ -$PKC_I\alpha$ binding.	0.01 $picoM^{-1}$ sec^{-1}
k_3	Dissociation rate constant C_1	1.0 sec^{-1}
k_4	Association rate constant C_1-DAG binding.	0.985 $picoM^{-1}$ sec^{-1}
k_5	Dissociation rate constant C_1^A	0.002 sec^{-1}
k_6	Rate constant for the activation of $PKC_I\alpha$	0.9981 sec^{-1}
k_0	Rate constant the deactivation of $PKC_{II}\alpha^A$	10 sec^{-1}
k_7	Association rate constant $DGK_I\zeta$ and $PKC_I\alpha^A$	0.981 $picoM^{-1}sec^{-1}$
k_8	Dissociation rate constant for C_2	0.002 sec^{-1}
k_9	Rate Constant for $DGK_I\zeta$ phosphorylation by $PKC_I\alpha^A$	0.9781 sec^{-1}
k_{10}	Association rate constant of C_1 with DAG	130.922 $picoM^{-1}sec^{-1}$
k_{11}	Dissociation rate constant for C_3	600 sec^{-1}
k_{12}	Rate Constant of DAG Phosphrylation	30.995 sec^{-1}
k_{13}	Rate constant of dephosphorylation of DAG_P	0.9825 sec^{-1}
k_{14}	Rate constant of DAG_P conversion to P.A	1.0 sec^{-1}
k_{15}	Rate constant of $DGK_I\zeta_P$ dephosphorylation	0.9981 sec^{-1}
λ_0	Translocation rate of $PKC_{II}\alpha$ from cytosol to plasma membrane	(0.0005* DAG) sec^{-1}
λ_{00}	Re-translocation rate of $PKC_I\alpha$ from plasma membrane to cytosol	0.01sec^{-1}
λ_5	Translocation rate of $DGK_{II}\zeta$ from cytosol to plasma membrane	(0.00005* DAG) sec^{-1}
λ_{55}	Re-translocation rate of $DGK_I\zeta$ from plasma membrane to cytosol	0.0085 sec^{-1}
λ_3	Re-translocation rate of $PKC_I\alpha^A$ from plasma membrane to cytosol	1sec^{-1}
λ_4	Degradation rate of $PKC_{II}\alpha^A$	0.001sec^{-1}

Supplementary Material 3: Differential equations describing the Gαq-induced local DAG-PKCα-DGKζ signaling.

$$\frac{d[DAG]}{dt} = k_1 * S_1 - k_4 * DAG * C_1 + k_5 * C_1^A - k_{10} * C_1 * DAG + k_{11} * C_3 + k_{13} * DAG_P * P \quad (17)$$

$$\frac{d[DAG_P]}{dt} = k_{12} * C_3 - k_{13} * DAG_P * P - k_{14} * DAG_P \quad (18)$$

$$\frac{d[C_1]}{dt} = k_2 * DGK\zeta * PKC_I\alpha - k_3 * C_1 - k_4 * C_1 * DAG + k_5 * C1^A \quad (19)$$

$$\frac{d[C_1]^A}{dt} = k_4 * DAG * C1 - k_5 * C_1^A - k_6 * C_1^A \quad (20)$$

$$\frac{d[PKC_I\alpha]}{dt} = -k_2 * DGK\zeta * PKC_I\alpha + k_3 * C_1 + \lambda_0 * PKC_{II}\alpha \quad (21)$$

$$\frac{d[PKC_I\alpha]}{dt} = k_6 * C_1{}^A + k_8 * C_2 + k_9 * C_2 - \lambda_3 * PKC_I\alpha^A - k_7 * DGK\zeta * PKC_I\alpha^A \quad (22)$$

$$\frac{d[PKC_{II}\alpha]^A}{dt} = \lambda_3 * PKC_I\alpha^A - \lambda_4 * PKC_{II}\alpha^A - k_0 * PKC_{II}\alpha^A \quad (23)$$

$$\frac{d[PKC_{II}\alpha]}{dt} = -\lambda_0 * PKC_{II}\alpha^A + k_0 * PKC_{II}\alpha^A \quad (24)$$

$$\frac{d[DGK\zeta_P]}{dt} = -k_{15} * DGK\zeta_P * P + k_9 * C_2 \quad (25)$$

$$\frac{d[C_2]}{dt} = k_7 * DGK\zeta * PKC_I\alpha^A - k_8 * C_2 - k_9 * C_2 \quad (26)$$

$$\frac{d[C_3]}{dt} = k_{10} * C_1 * DAG - k_{11} * C_3 - k_{12} * C_3 \quad (27)$$

$$\frac{d[DGK\zeta]}{dt} = -k_2 * DGK\zeta * PKC_I\alpha - k_7 * DGK\zeta * PKC_I\alpha^A + k_8 * C_2 + k_3 * C_1 + k_6 * C_1{}^A + k15 * DGK\zeta_P * P \quad (28)$$
